# A snail in the long tail: a new *Plekocheilus* species collected by the ‘Comisión Científica del Pacífico’ (Mollusca, Gastropoda, Amphibulimidae)

**DOI:** 10.3897/zookeys.516.10228

**Published:** 2015-08-10

**Authors:** Abraham S. H. Breure, Rafael Araujo

**Affiliations:** 1 Naturalis Biodiversity Center, P.O. Box 9517, 2300 RA Leiden, the Netherlands; 2 Museo Nacional de Ciencias Naturales (C.S.I.C.), c/ José Gutiérrez Abascal 2, 28006 Madrid, Spain

**Keywords:** Ecuador, Orthalicoidea, historical collection

## Abstract

Among the historical collection gathered by the ‘Comisión Científica del Pacífico’ during 1862–1865, type material was found of one of the species described on the basis of the material collected shortly afterwards. Inspection of the types revealed that only one specimen may be considered as type material of *Bulimus aristaceus* Crosse, 1869; this specimen is now designated as the lectotype. The other specimens are described as a new species, Plekocheilus (Plekocheilus) cecepeus.

## Introduction

Natural museum collections act as reservoirs of potential new species (Green 1998), but historical collections with the results of large expeditions during e.g. the 19th century, have usually been worked upon and new species have already been described. The expeditions of the ‘Comisión Científica del Pacífico’ (CCP) in South America during 1862–1865 (López-Ocón and Ponsati 2015), resulted in a large amount of specimens which are now in the collection of the Museo Nacional de Ciencias Naturales (MNCN) in Madrid. Hidalgo (1870, 1872) investigated the CCP-material and distributed part of it to colleagues, i.e. Crosse, Pfeiffer and Philippi. In total they described 20 new species of land shells.

One of these new species was *Bulimus aristaceus* Crosse, 1869 described from “Quito, reipublicae Aequatoris”, now placed in the genus *Plekocheilus* Guilding, 1828. Crosse (1869) first gave a brief diagnosis of this species, and later (Crosse 1870) published a description and a figure; both papers appeared in the Journal de Conchyliologie. Since Crosse had the habit of asking for types which had been described in that journal, the location of the type material of this taxon might have been expected in that journal’s collection (now part of the Paris museum). However, this material was not listed by Fischer-Piette (1950), and the types had not been traced when Breure (1979) listed all taxa in the genus *Plekocheilus*. While working on a new compilation the type material was found in the Madrid museum, but proved to consist of two different species, one of which is undescribed.

Recently Fontaine et al. (2012) studied the ‘shelf life’ between discovery and description of new species, based on a random selection of species described in 2007 from all kingdoms of life. They concluded that the avarage ‘shelf life’ was 20.7 years, ranging between zero and 206 years. While no specific data on molluscs were presented, the group ‘other invertebrates’ had an average ‘shelf life’ of ca. 15 years. Anderson (2004) coined the term ‘long tail’ for obscure products that form a niche market provided that they are available online. By analogy, one might call the undescribed species that remain on the museum shelves, well beyond the average time needed for a formal description, as being ‘in the long tail’.

We will re-describe the material of Crosse’s taxon found in the Madrid collection, and provide a description for the hitherto unrecognized species that was part of the material collected by the ‘Comisión Científica del Pacífico’.

## Methods

The following abbreviations are used in the text to refer to shell dimensions (in mm with an accuracy of 0.1 mm): D—diameter, H—shell height, HA—height of aperture, LW—height of last whorl, W—number of whorls, WA—width of aperture.

## Systematiscs

### Superfamily Orthalicoidea Albers, 1860 Family Amphibulimidae P. Fischer, 1873

#### Genus Plekocheilus

Taxon classificationAnimaliaStylommatophoraAmphibulimidae

Guilding, 1828

Plekocheilus Guilding 1828: 532.

##### Type species.

*Caprella undulata* Guilding, 1824, by monotypy.

##### Distribution.

West Indies, Panama, Colombia, Ecuador, Peru, Bolivia, Brazil, French Guyane, Suriname, Guyana, Venezuela.

#### Subgenus Plekocheilus (Eurytus)

Taxon classificationAnimaliaStylommatophoraAmphibulimidae

Albers, 1850

Eurytus Albers 1850: 169.

##### Type species.

*Helix pentadina* d’Orbigny, 1835, by subsequent designation (Albers 1860: 195).

##### Distribution.

West Indies (St. Lucia, St. Vincent), Panama, Venezuela, Brazil, Bolivia, Peru, Ecuador, Colombia.

#### Plekocheilus (Eurytus) aristaceus

Taxon classificationAnimaliaStylommatophoraAmphibulimidae

(Crosse, 1869)

Fig. 1

Bulimus aristaceus Crosse 1869: 185; Hidalgo 1870: 54, pl. 6 fig. 5; Crosse 1870: 105, pl. 6 fig. 5; Crosse 1871: 318; Pfeiffer 1877: 44; Miller 1878: 182; Paetel 1889: 208.
Eurytus aristaceus ; Pfeiffer and Clessin 1879 [1879–1881]: 227; Cousin 1887: 204.
Bulimulus aristaceus ; Paetel 1889: 221.
Plekocheilus aristaceus ; Pilsbry 1895 [1895–1896]: 88, pl. 4 fig. 4; Pilsbry 1902: xix; Richardson 1995: 302.
Plekocheilus (Eurytus) aristaceus ; Breure 1979: 29; Breure and Borrero 2008: 5.

##### Type locality.

“Quito, republica Aequatoris”.

##### Type material.

MNCN 15.05/7180, lectotype.

##### Re-description.

Shell 2.12 times as high as wide, with nearly covered, rimate perforation, ventricose-ovate, sides of the short spire slightly convex, moderately solid. Colour light chestnut-brown, with irregular blotches of reddish-brown, especially on last whorl which also shows a faint pattern of spiral bands, the interstices about as wide, which is due to the surface sculpture. Upper whorls slightly paler. Surface somewhat shining, finely granulate, entire teleoconch with a faint pattern of spiral lines of dot-like granulation, crossed by irregular longitudinal growth striae, especially on last whorl. Protoconch smooth and polished (eroded). Whorls 4.3, slightly convex, penultimate more convex and last whorl inflated, its height 0.93 total shell height. Suture well impressed, deeply descending in front. Aperture elongate-ovate, whitish inside, 1.80 times as long as wide, height 0.60 times shell height. Peristome hardly expanded, and slightly reflexed, whitish. Columellar margin slightly curved, above narrowly dilated at insertion to parietal wall, which has a thin, whitish callus.

Dimensions in mm: H 48.3, D 22.7, HA 29.1, WA 16.1, LW 44.8, 4.3 whorls.

**Figure 1.**
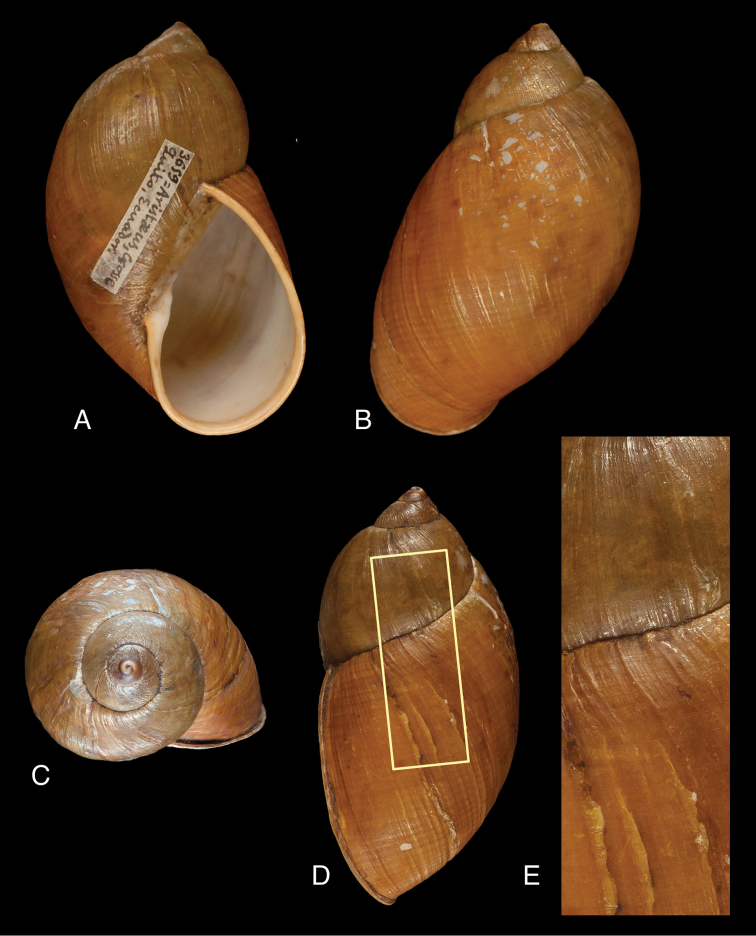
Plekocheilus (Eurytus) aristaceus (Crosse, 1869), lectotype MNCN 15.05/7180 (H = 48.3).

##### Remarks.

In the MNCN three lots are labelled ‘*Bulimus aristaceus* Crosse’ which are considered as syntypes. These lots appear not to be conspecific, and only lot MNCN 15.05/7180 is considered as type material of this taxon. As Crosse did not state on how many specimens his description was based, but no material of this species is present in the Paris collection, the sole specimen is now designated lectotype (**design. n.**) to fixate the taxon. The figure of Crosse (1870) does not entirely adequately represent the current state of the shell as the colour marks may largely have faded away. Crosse (1871) mentioned a variety, collected “Route de Quito à Napo (prof. Orton)”; this would indicate that this species might occur on the eastern slopes of the Cordillera, but we have not seen this material.

#### Subgenus Plekocheilus (Plekocheilus)

Taxon classificationAnimaliaStylommatophoraAmphibulimidae

Guilding, 1828

##### Distribution.

West Indies (St. Vincent, Barbados), Venezuela, Ecuador, Colombia.

#### Plekocheilus (Plekocheilus) cecepeus sp. n.

Taxon classificationAnimaliaStylommatophoraAmphibulimidae

http://zoobank.org/5546825B-CF3D-4056-8778-AB58D5824937

Fig. 2

##### Diagnosis.

A moderately small species of Plekocheilus (Plekocheilus), characterized by the irregularly shaped, widely spaced, narrowly reddish-brown axial colour streaks, and the spiral series of oblong granules between the axial riblets, becoming a malleate pattern on the dorsal side of the last whorl.

##### Description.

Shell up to 45.0 mm, 1.76 times as high as wide, imperforate, ovate, sides of spire hardly convex, moderately solid. Colour light chestnut-brown, with irregular, axial streaks of reddish-brown, partly as oblique lines or as zig-zag lightning streaks and partly broken up. Upper whorls paler or denuded of epidermis. Surface somewhat glossy, with moderately strong axial riblets, partly broken up into smaller ones, especially on lower ventral part of last whorl; penultimate whorl with spirally arranged oblong granules in between axial riblets, becoming gradually stronger and forming, on dorsal side of last whorl, a malleated pattern of spirally arranged, broken axial riblets partly irregularly shaped. Protoconch smooth (eroded). Whorls up to 4.9, hardly convex, last whorl 0.94 times shell height, somewhat swollen. Suture well impressed, descending in front and abrubtly ascending behind lip. Aperture elongate-ovate, 2.28 times as long as wide, height 0.72 times shell height. Peristome expanded and reflexed, whitish or pinkish, slightly curved at insertion to parietal wall. Columellar margin curved, above a more or less weak fold entering the aperture; broadly dilated above at the insertion to the parietal wall, which has a thin whitish or translucent, broadly spreading callus.

Dimensions in mm: H 37.8–45.0, D 22.3–25.4, HA 23.5–32.2, WA 11.6–14.8, LW 34.0–42.2, 3.8–4.9 whorls. Holotype H 44.8, D 25.4, HA 32.2, WA 14.1, LW 42.2, 4.8 whorls.

**Figure 2.**
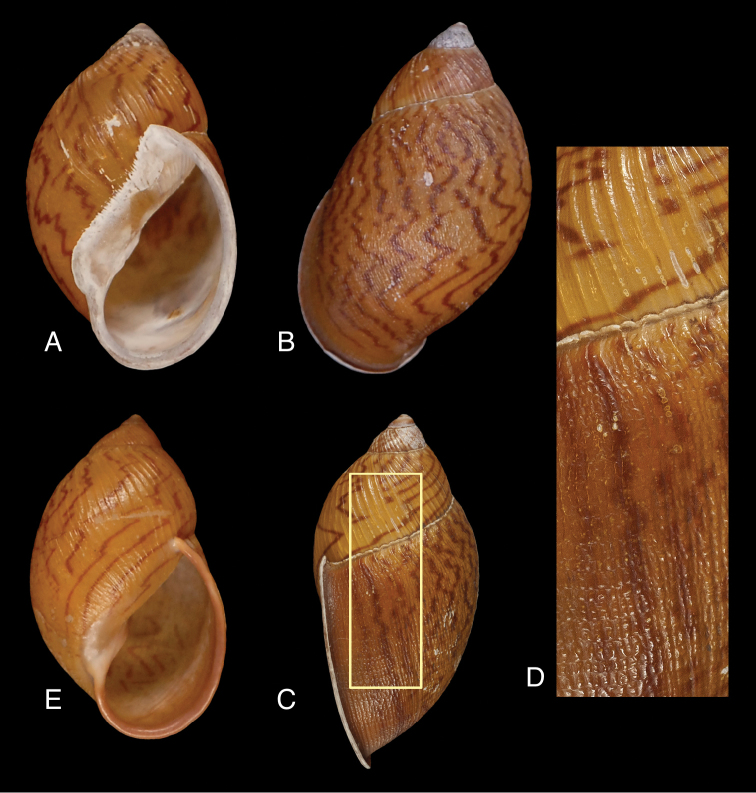
Plekocheilus (Plekocheilus) cecepeus sp. n., **A–D** holotype MNCN 15.05/60013H (H = 44.8) **E** paratype MNCN 15.05/60013P (H = 38.8).

##### Type locality.

Ecuador, “Quito” (teste Hidalgo 1893: 102). See remarks.

##### Type material.

MNCN 15.05/60013H, holotype; MNCN 15.05/60013P, five paratypes; MNCN 15.05/7477P, three paratypes.

##### Comparison with other species.

This new species bears resemblance with Plekocheilus (Plekocheilus) blainvilleanus (Pfeiffer, 1848) from northern Venezuela, but differs in being smaller, the last whorl less malleated, and having the suture abruptly ascending behind the lip. It may also be compared to the Venezuelan Plekocheilus (Plekocheilus) fulminans (Nyst, 1843), from which it differs by having a larger aperture, the lip less thick, and a less pronounced fold in the columella. Finally, it resembles Plekocheilus (Plekocheilus) alticola Haas, 1955 from Venezuelan Guayana, but differs by being slightly larger, and having a less malleated sculpture.

##### Remarks.

The type locality is unfortunately very imprecise, which was not uncommon with material collected during the 19th century (Breure and Borrero 2008). Almagro (1866: 81) briefly described the two months the expedition stayed in Quito from the beginning of December 1864 to the beginning of 1865. During that period they made excursions in the province of Imbabura and to the volcans of Antisana, and Pichincha. A detailed list of localities of Ecuadorian material, with collectors and number of specimens can be found in Almagro 1866: 163–164. However, it cannot be excluded that the material was actually colected at a considerable distance from the capital, and it remains to be seen if future collecting may provide more precise localities for this species.

##### Etymology.

The specific epithet is formed after the abbreviation for the ‘Comisión Científica del Pacífico’ (CCP). Named in honour of the expedition members of this commission, i.e. Patricio María Paz y Membiela (1808–1874), Manuel Almagro (1834–1895), Fernando Amor (1820–1863), Francisco de Paula Martínez y Sáez (1835–1898), Marcos Jiménez de la Espada (1831–1898), Rafael Castro y Ordóñez (1834–1865), and Juan Isern (1825–1866). 150 years ago they returned with many undescribed species and this novelty remained all those years on the shelves. The epithet is used as a noun.

## Discussion

López-Ocón and Badía (2003) have discussed the political-historical context of this expedition and have described the up’s and down’s in the study of the collected material. Although description of new species that have remained unnoticed for more than a century remains a rare event (but see e.g., Breure 2011: 44–45), it highlights the need for revisions of museum collections by specialists, and especially the historical parts of these. This rare find also stresses the need for additional field work in the area, trying to locate the true home of this novelty.

## Supplementary Material

XML Treatment for Plekocheilus

XML Treatment for Plekocheilus (Eurytus)

XML Treatment for Plekocheilus (Eurytus) aristaceus

XML Treatment for Plekocheilus (Plekocheilus)

XML Treatment for Plekocheilus (Plekocheilus) cecepeus
